# Food Safety Governance and Guardianship: The Role of the Private Sector in Addressing the EU Ethylene Oxide Incident

**DOI:** 10.3390/foods11020204

**Published:** 2022-01-12

**Authors:** Aleksandra Kowalska, Louise Manning

**Affiliations:** 1 Institute of Economics and Finance, Maria Curie-Skłodowska University, pl. Marii Curie-Skłodowskiej 5, 20-031 Lublin, Poland; aleksandra.kowalska@mail.umcs.pl; 2 School of Agriculture, Food and the Environment, Royal Agricultural University, Stroud Road, Cirencester GL7 6JS, UK

**Keywords:** foods not of animal origin, food safety governance, ethylene oxide, sesame, surveillance, *Salmonella*, hybrid governance, reflexive governance

## Abstract

Sesame seeds within the European Union (EU) are classified as foods not of animal origin. Two food safety issues associated with sesame seeds have emerged in recent years, i.e., *Salmonella* contamination and the presence of ethylene oxide. Fumigation with ethylene oxide to reduce *Salmonella* in seeds and spices is not approved in the EU, so its presence in sesame seeds from India was a sentinel incident sparking multiple trans-European product recalls between 2020–2021. Following an interpretivist approach, this study utilises academic and grey sources including data from the EU Rapid Alert System for Food and Feed (RASFF) database to inform a critical appraisal of current EU foods not of animal origin legislation and associated governance structures and surveillance programs. This is of particular importance as consumers are encouraged towards plant-based diets. This study shows the importance of collaborative governance utilizing data from company testing and audits as well as official regulatory controls to define the depth and breadth of a given incident in Europe. The development of reflexive governance supported by the newest technology (e.g., blockchain) might be of value in public–private models of food safety governance. This study contributes to the literature on the adoption of risk-based food safety regulation and the associated hybrid public–private models of food safety governance where both regulators and private organizations play a vital role in assuring public health.

## 1. Introduction

Effective food safety governance is pivotal to public health [1]. Thus, the first general objective of European Union (EU) food law is to guarantee a high level of protection of human life, health, and consumers’ interests. Private sector practices to reduce the risk of a food safety incidents include third-party certification, public–private partnerships, and voluntary standards. These are often defined in supply chain specifications and can work within the market, independently of regulation, or can be considered and integrated when regulators are developing risk-based regulation. Food companies seek to establish the most effective risk-based monitoring programs as they are responsible for the safety of the products they sell and controlling any food safety issues which arise that may have a negative impact on public health and their competitive position. Furthermore, risk-based monitoring is increasingly required in EU food law, e.g., Regulation (EU) 2017/625 [2]. The public–private partnerships between regulators and industry provide a hybrid model of food safety governance. Hybridization covers the ‘who’ (public and private actors) of regulation, the ‘how’ (command-and-control vs. responsive regulation), the ‘where’ (global, local, and intermediate level), and the ‘what’ (business vs. social regulation) of policy initiatives [3,4].

Hybridization is driven through innovation in food governance structures [5], i.e., public–private partnerships can deliver innovative, agile, and flexible practices and governance activities when compared to purely regulatory governance approaches [6]. In a hybrid public–private partnership approach to food safety governance, private market approaches can be integrated with public regulatory controls. However, there are some challenges, as the perceptions and interests of the involved actors may vary, and so collaborative processes need to be coordinated to meet mutual and independent goals. Efficient communication, including rapid and sufficient exchange of information between public and private institutions, is difficult to achieve in practice; parties may have different expertise or resources (financial, human, social, and physical) and self-interest of both private and public entities can hinder cooperation. Based on the Guidelines for Successful Public–Private Partnerships [7], four principal private sector provisioning roles are identified in such approaches. These are provisions of additional capital and resources as a partnership, e.g., product testing facilities; alternative management and implementation skills which can be mutually strengthening; adding value to consumers and the public at large; and better identification of needs leading to optimal use of resources. To achieve this, effective data sharing and exchange is required between public and private actors, and the process must be agile and responsive to changes in risk [8].

The safety and legality of plant-based foods is of particular interest [9], as they are promoted as key elements of sustainable diets and as a result consumption is likely to increase, either in the raw or processed state. The EAT-Lancet Commission provides guidance for the necessary shift towards healthy and sustainable diets, which consists of increasing consumption of plant-based foods and substantially reducing consumption of animal sourced foods [10]. The consensus definition of “sustainable diets” was proposed at the International Scientific Symposium “Biodiversity and Sustainable Diets: United Against Hunger” held at FAO, in Rome, in 2010, as follows:

*Sustainable Diets are those diets with low environmental impacts which contribute to food and nutrition security and to healthy life for present and future generations. Sustainable diets are protective and respectful of biodiversity and ecosystems, culturally acceptable, accessible, economically fair and affordable; nutritionally adequate, safe and healthy; while optimizing natural and human resources*

([11], p. 7).

Hence, the four principal domains of sustainable diets are health, economics, society, and the environment [12]. This places particular focus on food safety regulation associated with foods not of animal origin, especially if they are being proposed as forming a greater proportion of the human diet in the future. Within the EU, foods are differentiated in terms of official controls as foods of animal origin and foods of non-animal origin. Foods of non-animal origin that are imported into the EU from third countries are subject to official controls legislation where the controls at border inspection points may increase if the risk associated with a certain food safety hazard is deemed higher at a particular point in time by the regulatory authorities [13]. The original legislation lays down the general requirements of food law in the EU. Food business operators should advise when there are imports of foods into the EU and complete the common entry document (CED) which contributes to the effective functioning of food traceability system, information exchange, and risk management. Regulation (EC) No 882/2004 establishes a harmonized framework of general rules for the organization of official controls in the EU. The frequency of sampling of foods of non-animal origin at the point of entry into the EU is based on known public health concerns or emerging changes in risk rating that requires increased levels of official controls. These sampling protocols are detailed in Annex I of the regulation. The relevant legislation to consider changes in sampling protocols is the Commission Regulation (EC) No 669/2009, Commission Implementing Regulation (EU) No 884/2014, Commission Implementing Regulation (EU) 2015/175, Commission Implementing Regulation (EU) 2017/186, Commission Implementing Regulation (EU) No 2018/1660, and Commission Implementing Regulation (EU) 2019/1793 and then as amended by Commission Implementing Regulation (EU) 2020/625 [14,15,16,17,18,19,20]. This ongoing iteration of changes to the official control systems demonstrates the regular need to update sampling plans for plant derived materials imported into the EU as their risk status changes.

Following an interpretivist approach, this study uses academic and grey sources (including secondary data from the EU Rapid Alert System for Food and Feed or RASFF) database. These sources inform a critical appraisal of food safety issues related to EU foods of non-animal origin legislation, and the associated collaborative food governance structures and surveillance programs that identify and seek to mitigate such issues. The role of official controls and the organizations’ own food safety testing regimes are of particular importance within this collaborative food safety surveillance system.

Effective food safety guardianship (regulations, enforcement, and surveillance systems) by regulators and market actors reduces the likelihood of food safety incidents occurring [21]. Guardians, in this context, monitor and protect food, processes, consumers, individual organizations, supply chains, and nations against illegal activity [22], irrespective of whether such activity is an intentional or an unintentional action. Whilst the term guardian has emerged from the food fraud literature, it is considered in this study in a wider application within food safety governance. Guardianship is considered here as the process of defending and securing something, e.g., the integrity of a food product or trust in a food supply chain standard. The term also includes the overarching regulatory protection applied where an individual or business is unable to protect themselves or has insufficient information or empowerment to make decisions on their own behalf. The absence of capable food guardians, or wider guardianship processes, is a key factor in the defence against, or conversely actualization of, failures in food supply chains [23]. Whether these guardians operate in the private sector (certification companies, quality assurance, technical departments, and “whistleblowers”) or in the public sector (government or public health inspectors), they all collectively play a collaborative role in delivering safe and legal food consistently to consumers/citizens [24]. Despite the continuous improvement of food safety governance and food safety guardianship over time, in the past decade, several food product categories have been associated with public health incidents and foodborne outbreaks worldwide, resulting in significant health and economic losses [25,26,27].

Sesame seeds are the commodity that is considered in this research, a product subject to major recalls that led to significant supply chain disruption in 2020/21. The safety of plant derived foods, especially regarding cumulative, persistent food safety hazards is of interest here. This paper considers the role of public and private governance in this context and the role of sentinel signals in such governance processes, particularly private company testing.

## 2. Literature Review

India was the third largest producer of sesame seeds (*Sesamum indicum*) worldwide in 2018 (746 thousand tons), following Sudan and Myanmar [28]. In this time, India was the second largest exporter of sesame seeds globally with almost 44% of the production volume being exported (326.8 thousand tons) [29]. In 2018, the import quantity of sesame seeds to the EU was 166 thousand tons with 68 thousand tons specifically imported from India [28]. Thus, every fifth ton of sesame seeds exported from India entered the EU food market, which made the EU a strategic trading partner. Sesame seeds, a rich source of lipids, fatty acids, endogenous antioxidants, proteins, carbohydrates, and minerals, are gaining in popularity as a raw ready to eat food [30,31], as well as a component ingredient of other foods, especially bakery products. Their important phyto-constituents are used in traditional and modern systems of medicine including wound healing, hepatic problems, memory disturbances, autoimmune encephalomyelitis, atherosclerosis, cancer, and hypertension [32].

Tahini is a high fat sesame paste produced from dehulled and roasted or unroasted sesame seeds. It is often used as an ingredient in other food products, for example, salad dressing, baba ghanoush, helva, and hummus [31,33,34,35,36,37,38,39,40,41]. However, it is a product linked with recalls due to sesame seed contamination with *Salmonella* [30,31,42,43,44], and there are multiple instances of *Salmonella* contamination. Indeed, some studies show tahini allows *Salmonella* to survive for at least 16 weeks [31]. Pathogenic contamination of tahini is an issue as the high fat content allows such bacteria to survive [36]. Foodborne outbreaks of Salmonellosis are associated with low water activity products such as sesame seeds across the world and examples are collated in Table 1.

Contamination of sesame seeds with *Salmonella* is an ever-present issue in the EU, with 658 notifications in the RASFF database between 2001–2020 [66]. Contamination with *Salmonella* can occur with sesame seeds during growth, storage, post-harvest processing (de-hulling, roasting, grinding, and drying), cross-contamination, any instance of poor hygiene conditions, and via irrigation water, soil, manure, wild birds, rodents, and other animals [30,31,67]. Thus, good hygienic practices through the whole supply chain to prevent cross contamination are essential. Roasting seeds at 110–150 °C can also inactivate *Salmonella* [31]. Measures to reduce the viability of *Salmonella* serovars in commercial tahini and products containing hydrated tahini include the use of acetic and citric acid in ready to eat foods [36]. As it has a mutagenic action on the bacteria, gaseous ethylene oxide is a chemical fumigant, effective at inactivating *Salmonella* spp. [68,69], in seeds and spices including cumin seeds [70,71] and black pepper [72].

The European Chemicals Agency (ECHA) define ethylene oxide as a chronic food safety hazard that is a carcinogen, a mutagen, and toxic to reproduction [73]. Any use of ethylene oxide as an active substance in a crop protection product is not approved in the EU due to its harmful nature, and a maximum residue limit (MRL) of 0.05 mg/kg is set for sesame seeds that are sold within the EU [74]. However, the use of ethylene oxide as an antimicrobial/crop protection product is approved in some parts of the world, and Codex Alimentarius has set no MRLs for ethylene oxide, the United States of America (USA) sets an MRL of 7 mg/kg, and Canada has been consulting on the MRL that should be agreed for sesame seeds [75,76]. Ethylene oxide is currently undergoing a registration review in the USA and the draft risk assessment contains a recommendation to remove the MRL for ethylene oxide [77].

The need for exporters to the EU to reduce *Salmonella* levels in sesame seeds and to have levels of ethylene oxide below the stated MRL is a prerequisite to access the European market. Failure to do this means that the consignments will be rejected either if sampled at EU border inspection points, via other forms of official controls or by the companies themselves as part of their supplier assurance program. The frequency of purposive official controls sampling for foods originating from specific countries depends on the regulator’s assessment of a given food safety issue and the associated pre-determined risk to public health [13]. Thus, the presence of *Salmonella* or ethylene oxide as a contaminant of sesame seeds is an example of a grey swan, i.e., it is a food safety risk that is knowable, assessable, and can be mitigated for, even eliminated, with appropriate official controls and business level controls in place [78,79,80].

Recognition of the *Salmonella* concerns associated with sesame seeds means they are on the list of products subject to extra regulatory surveillance checks. Commission Implementing Regulation (EU) 2019/1793 of 22 October 2019 called for an increased frequency of regulatory controls to be carried out for the presence of *Salmonella* in sesame seeds originating from India (20% of all consignments) and sesame seeds originating from Ethiopia, Nigeria, Sudan, and Uganda (50% of all consignments) [15]. In July and January 2017, sesame seeds from Sudan and Uganda were subject to an increased level of official controls for *Salmonella* and were required to be accompanied by a certificate stating *Salmonella* was absent in 25 g [15].

Through exchanging data regarding non-compliances, RASFF notifications are initiated by border inspection points or via the activities of national food protection agencies to address a specific food incident or issue [13,81]. In September 2020, a series of product recalls began for the presence of an unauthorized substance, ethylene oxide, across the EU for sesame seeds originating from India or products that contained sesame seeds from India as ingredients. These notifications were ex-post reactive measures designed to protect public health, and to identify any potential violation of agri-food chain legislation. The first RASFF notification on 9 September 2020 arose from an organization’s assurance processes with the notification stating the presence of up to 186 mg/kg of ethylene oxide in sesame seeds, 3700 times higher than the MRL. The organization could have implemented either existing or reactive product testing or planned or unannounced supply chain audits; this was not disclosed within the RASFF notification. A further thirty-one notifications were posted onto the RASFF database between 9 September and 22 October 2020 when Commission Implementing Regulation (EU) 2020/1540 was adopted regarding sesame seeds originating from India [82] (Table 2).

Commission Implementing Regulation (EU) 2020/1540 of 22 October 2020 amending Commission Implementing Regulation (EU) 2019/1793 highlighted concerns with sesame seeds originating in India and the presence of ethylene oxide contamination [82]. The sampling rate for sesame seeds was set at 50% for pesticide residues (Table 2). This regulation came into force on the 25 October 2020. The regulation notes that: “In September 2020, very high levels of ethylene oxide were notified through the RASFF as regards certain batches of sesame seeds originating in or consigned from India and having entered the Union. Those levels are exceeding more than 1000 times the maximum residue level of 0.05 mg/kg applicable for ethylene oxide in accordance with Regulation (EC) No 396/2005 of the European Parliament and of the Council. Such contamination constitutes a serious risk to human health within the Union, as ethylene oxide is classified as a mutagen, category 1B, a carcinogen, category 1B, and a reproductive toxicant, category 1B, in accordance with Regulation (EC) No 1272/2008 of the European Parliament and of the Council. Moreover, ethylene oxide is not approved as an active substance for use in plant protection products in the Union.”

The implementing Regulation (EU) 2020/1540 requires that India do prior testing of sesame seeds intended for export to the EU, to certify compliance with MRL of pesticides applicable for ethylene oxide [82]. Testing results must be confirmed by an official certificate, which must accompany all consignments [83]. The provisions of this regulation create a foundation for collaborative food safety governance between India and the EU. The study approach for this research is now explained.

## 3. Materials and Methods

The methodological approach to explore this incident is composed of three phases with phase 1 being the foundational literature search. Phase 2 was the analysis of the secondary data to develop a synthesized series of sesame related *Salmonella* incidents held in regulatory databases and publicly available literature. Phase 3 was the detailed data collection and analysis of the notifications held within the publicly available section of the RASFF database with particular focus on India as a country of origin. The following research question was considered:

RQ1. What is the footprint of the ethylene oxide incident and what was the role of the ‘company’s own checks’ in the food safety governance processes in the 2020–2021 incident?

Phase 1

We searched the following databases: Science Direct, Google Scholar, and Google (to include grey literature) to frame the research. The key search terms are shown in Table 3. The terms were used in a series of combinations, i.e., through an iterative literature review method. Iterative literature review is grounded by a foundational literature search using a series of iterative searches. In undertaking the searches for a given combination of search terms, the first 100 items in each search are considered for relevancy and any duplication [13]. All relevant papers were collated and the titles and abstracts read. The papers were then read in full (*n* = 12) and screened for relevance and value in supporting the discursive narrative and argument in this paper.

Phase 2

Analysis of secondary data for the synthesized series of sesame related *Salmonella* incidents (Table 1) was generated from the iterative literature search with the addition of searches for product recalls between 2019 and 2021 connected with *Salmonella* and sesame seed related products. The databases searched were the following national regulator websites: Canadian Food Inspection Agency (CFIA), US Food and Drug Administration (FDA), Food Safety Authority of Ireland (FSAI), Food Standards Agency (FSA). and Product Safety Australia (PSA). The RASFF database [66] was considered specifically in the next phase of the methodological approach.

Phase 3

The product category ‘nuts, nut products and seeds’ on the RASFF database was searched for total notifications. There were 10,244 notifications for ‘nuts, nut products and seeds’ between 1 January 2004 and 31 August 2021. One in five notifications within the RASFF in this time period were within this product category [66]. The high level of notifications is partly due to the aforementioned purposive sampling associated with EU legislation which provides for an increased frequency of regulatory controls for certain food products at border inspection points [13].

The RASFF notification ‘type’ is determined by three fields in the database. These categories are (1) product type (food, feed, or food contact material), (2) notification classification (alert, information, or border rejection), and (3) notification basis, indicating what type of control, report, or investigation was the basis of the notification (border control, official control on the market, company internal-check, consumer complaint, or food poisoning) [66]. A notification is classified as an ‘alert’ and is triggered when the food, feed, or food contact material presents a serious risk on the market and rapid action is or might be required, generally aimed at withdrawing the product from the market. An ‘information notification’ concerns a food that does not require rapid action, either as the product is not on the market at the time of the report or the public health risk is determined as being low. A ‘border rejection notification’ is created when a foodstuff is prevented from entering the EU as it is considered a risk to food or feed safety [9,13,66,84]. The main dataset obtained from the RASFF database came from the use of four search criteria: product country of origin (India), notification type (food), hazard category (pesticide residues), and date of notification (from 1 September 2020 to 31 August 2021). This meant the notifications regarding the presence of unauthorized ethylene oxide in products containing sesame seeds could be extracted.

## 4. Results

**Figure 1:**
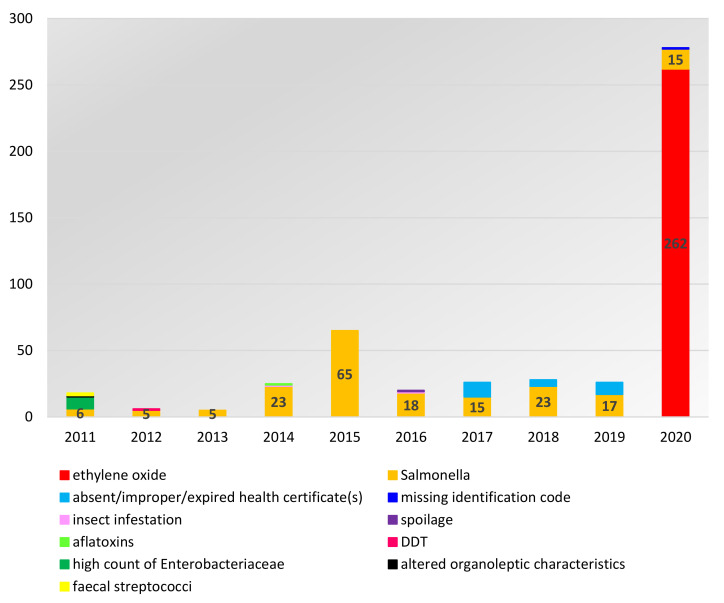
RASFF notifications linked to sesame seeds from India per substance/hazard over the period 1 January 2011–31 December 2020: the data broken down by year (Source: Own elaboration based on [66]).

**Figure 2:**
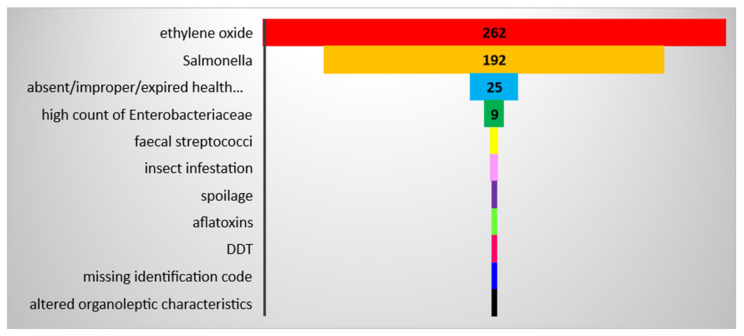
RASFF notifications linked to sesame seeds from India per substance/hazard over the period 1 January 2011–31 December 2020: the aggregated data at the period of analysis level (Source: Own elaboration based on [66]).

Notifications related to sesame seeds from India identified within the RASFF database over the period 2011–2020 included the presence of ethylene oxide (*n* = 262); and the presence of *Salmonella* (*n* = 192). Then, at a much lower frequency, absence of health certificate(s) or a certified analytical report or improper/expired health certificate(s), (*n* = 25); high counts of *Enterobacteriaceae* (*n* = 9); insect infestation (hazard category: foreign bodies) (n = 2); faecal *Streptococci* (hazard category: microbial contaminants (other)) (*n* = 2); spoilage or altered organoleptic characteristics (hazard category: organoleptic aspects) (*n* = 2); aflatoxins (hazard category: mycotoxins) (*n* = 1); prohibited substance Dichlorodiphenyltrichloroethane or DDT (hazard category: pesticide residues) (*n* = 1); and missing identification code (hazard category: labelling absent/incomplete/incorrect) (*n* = 1) (Figure 1 and Figure 2).

Sesame seeds and products thereof represented about 66% of the notifications for nut and seed products originating from India over the longer time period 1 January 2004–31 August 2021 (*n* = 642). Only 9.5% of the RASFF notifications in this time frame related to nuts, nut products, and seeds were associated with India as a country of origin (*n* = 977). Analysis of RASFF notifications associated with sesame seeds and products thereof from India demonstrates that the most common problem over the period 2012–2019 was *Salmonella* contamination (Figure 1).

An audit was carried out by the Directorate-General for Health and Food Safety in India from 23 to 27 October 2017 to assess the official control systems in place for seeds for human consumption with specific emphasis on sesame seeds that were processed for export into the EU [85]. This audit was a follow-up to a 2014 audit as there was particular concern about the level of RASFF notifications for *Salmonella* spp. associated with sesame seeds. The 2017 report highlighted a number of concerns, namely a lack of traceability back to farm and poor hygiene controls, especially with regard to bird control at multiple stages of the supply chains. These weak hygiene controls included sale of sesame seeds in open markets, where the sesame seeds are traded by small scale farmers, when the potential for birds to come into contact with the seeds in that environment is high. This is one example of how sesame seeds can be contaminated post-harvest, but at all stages of sesame seed growing, harvesting, aggregation, cleaning, and distribution there is the opportunity for microbiological contamination from multiple sources. This needs to be addressed in a specific *Salmonella* control plan, that is developed, implemented, monitored, and verified in line with the requirements therein. The recommendation was that where heat treatment processes are in place, they must be validated to demonstrate they have the capability to eliminate the potential for *Salmonella* spp. contamination of sesame seeds. However, there is always the concern of emergent heat resistant strains of *Salmonella*, where specific critical control points then lose their efficacy.

**Figure 3:**
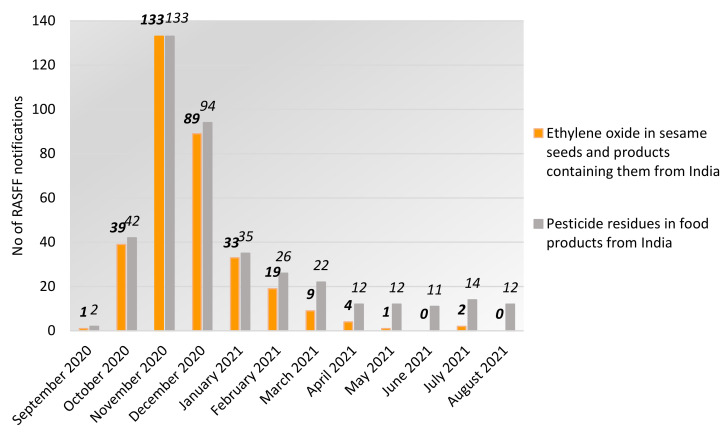
Food safety issues related to pesticide residues in food products from India notified to RASFF over the period September 2020–August 2021 (Source: Own elaboration based on [66]).

At the end of 2020, the ethylene oxide contamination of sesame seeds from India became an emergent pressing issue within the RASFF database as shown in Figure 1 and Figure 2. There were 262 RASFF notifications related to the presence of an unauthorized substance, ethylene oxide, in sesame seeds and products containing sesame from India in 2020 (type: food; hazard category: pesticide residues), i.e., 1 notification in September 2020, 39 notifications in October 2020, 133 notifications in November 2020, and 89 notifications in December 2020. It should be noted that the notifications have continued in 2021 but the number of notifications has been in decline since November 2020 (Figure 3). In fact, the presence of the unauthorized substance ethylene oxide in sesame seeds and products containing them was the major issue (87% of notifications) related to pesticide residues in food products originated from India between September 2020–February 2021. Again, it should be noted that this statistic was skewed by the purposive nature of sampling. There were 261 RASFF notifications for ethylene oxide contamination of Indian sesame seeds and products containing them (see Figure 3) and 3 RASFF notifications for ethylene oxide contamination of Indian food products other than sesame seeds and products containing them (amaranth (*n* = 2), psyllium husks (*n* = 1)) in the fourth quarter of 2020.

There were 61 RASFF notifications for ethylene oxide contamination of Indian sesame seeds and products containing them. There were 13 notifications for ethylene oxide contamination of Indian foods other than sesame products namely: (okra (*n* = 3), ginger and products thereof (*n* = 3), drumsticks (Moringa oleifera) (*n* = 2), curry powder (*n* = 1), turmeric (*n* = 1), amaranth (*n* = 1), onion granulates (*n* = 1), and dried shallots (*n* = 1)) in the first quarter of 2021.

Five RASFF notifications for ethylene oxide contamination of Indian sesame seeds and products containing them, and 26 such reported food safety incidents related to Indian foods other than sesame products were released in the second quarter of 2021. These were guar gum/flour (*n* = 5), psyllium (husks/flakes) (*n* = 3), ground ginger/ginger extract (*n* = 2), black pepper (*n* = 2), Ashwagandha (*n* = 2), fenugreek (*n* = 2), curcuma/curcumin (*n* = 2), amaranth (*n* = 1), curry powder (*n* = 1), dehydrated onions (*n* = 1), celery (*n* = 1), green coffee (*n* = 1), ground coriander seeds (*n* = 1), food supplements (*n* = 1), and linseed (*n* = 1). Thus, whilst the notifications regarding ethylene oxide contamination of foods of Indian provenance in 2020 were mainly associated with sesame seeds and products containing them, the notifications in 2021 were associated with a wider range of products of non-animal origin from India, while at this time, the problem of the presence of ethylene oxide in sesame seeds from India seems to no longer be a source of RASFF notifications, the ethylene oxide recalls have spread to other foods and food ingredients (including food additives). This shows a diverse public health problem with the presence of ethylene oxide in plant derived foods and food ingredients. More recently, in 2021 ethylene oxide has been identified in a range of additives and supplements including calcium carbonate, coconut milk powder, magnesium oxide, locust bean gum, guar gum, senna leaf extract, curry leaf extract, ginseng root, and xanthan gum, among others [66].

Considering the presence of unauthorized ethylene oxide in sesame seeds and products containing them shows India was the country of origin for over 90% of the notified products (*n =* 330) over the period September 2020 and August 2021. Other countries of origin were: Netherlands (*n* = 11), France (*n* = 5), Sweden (*n* = 3), Germany (*n* = 2), Czechia (*n* = 2), Italy (*n* = 1), Nigeria (*n* = 1), Ethiopia (*n* = 1), China (*n* = 1), the UK (*n* = 1), Paraguay (*n* = 1), Bosnia and Herzegovina (*n* = 1), Burkina Faso (*n* = 1), Denmark (*n* = 1), and Belgium (*n* = 1). This finding is as sesame seeds from India are sold as consignments of sesame seeds and used as an ingredient in a range of products including cereal and bakery products (e.g., bagels, burger buns, crackers, biscuits), salads, chocolate, sesame oil, and Asian dishes. This means the origin of the food that was part of the notification conflicted with the origin of the sesame seeds that came from India. About 57.3% of the incidents were notified by the Netherlands (*n* = 189), followed by Germany (*n* = 35), Greece (*n* = 13), Italy (*n* = 12), Belgium (*n* = 11), Czechia (*n* = 8), Norway (*n* = 6), and Austria (*n* = 6). Other countries had five notifications or less, being Croatia, Spain, Finland, Sweden, Luxemburg, Romania, Portugal, Switzerland, France, Slovenia, Denmark, Latvia, and Poland [66] which demonstrated the Europe-wide nature of the ethylene oxide incident.

Analysing more detailed RASFF data [66] within the period September 2020 and August 2021, regarding unauthorized ethylene oxide in sesame seeds shows that 99% of the cases were categorized as serious risk. Nearly 79% of the cases were alerts (almost entirely categorized as serious risk), when rapid action is or might be required in another country, whereas 18% of the cases were information for attention (also categorized as serious risk) which means an information notification is released related to a product that: (1) is present only in the notifying member country; or (2) has not been placed on the market; or (3) is no longer on the market [86]. It is worth emphasizing that the main source of information were the companies’ own checks (for 292 out of 330 analysed cases which account for 88.5% of the incidents concerned) which is unusual, as companies’ own checks are the basis of only 27.1% of all the RASFF notifications [66]. This is an important finding (see RQ1). Whilst the RASFF database routinely contains notifications from border inspection point checks, mainly from purposive sampling, the notifications in this case are also being driven by more responsive surveillance testing by organizations within the food supply chain, i.e., the organisations are actively involved in food safety guardianship. The ‘company’s own-checks’ have thus gained significant importance as triggers for RASFF notifications [66].

The most common actions carried out in connection with the RASSF notifications regarding the presence of ethylene oxide in food products containing sesame originating from India within the period from September 2020 to August 2021 were informing the consignor, informing the recipient(s), and withdrawal from the market (Table 4). Indeed only 18 notifications to date, including informing the authorities (*n* = 7) and official detention (*n* = 11), suggest a primary role by competent authorities. The total number of actions taken regarding the notifications of non-compliant sesame seeds/sesame related food products traceable to India as a source exceeds the overall number of the incidents, as in some cases several measures were taken for a single notification (Table 4). These measures might include informing consignor, then recall from consumer, public warning press release, withdrawal from recipient(s), or destruction.

Furthermore, the distribution of these products was wide, and numerous countries might have been involved in one RASFF notification either as the notifying country (n), country of origin (o), highlighted as a country where distribution has occurred (d), flagged for follow-up (ffup), or flagged for attention (ffa). One notification, for example, had the following footprint across global supply chains:

(o) India, (d) (n) (ffup) Netherlands, (ffup) Slovenia, (d) Australia, Belarus, Canada, Hong Kong, Jordan, Kuwait, Malaysia, Morocco, Qatar, Russia, Saudi Arabia, Singapore, South Africa, Ukraine, United Arab Emirates, UK, and USA, and (d) (ffup) Austria, Belgium, Croatia, Czechia, Denmark, Estonia, Finland, France, Germany, Hungary, Iceland, Ireland, Latvia, Lithuania, Norway, Poland, Portugal, Romania, Slovakia, Spain, Sweden, and Switzerland [66].

This incident demonstrates the role of companies undertaking their own checks as a key element of food safety governance. This means that additional due diligence checks, implemented as corrective actions by organizations in their role as guardians during the incident, was vital to ensure public health. One organization that implemented a withdrawal (Linwoods [87]) stated in their withdrawal notice (27 November 2020):

“All raw materials used in our products are rigorously tested at goods intake stage when we carry out lab testing for over 400 pesticides. Unfortunately, ethylene oxide was not highlighted as a potential pesticide by European Food Standards Agency to any food company in Europe until November 20 and therefore it was not on Linwoods’ lab testing schedule”.

[87]

This recall notice suggests that, prior to the incident, ethylene oxide was not recognized as a food safety risk of concern at supply chain level, as regulations banned its use and as a result it was a ‘given’ that it would not be present, i.e., incoming food material was expected to comply with legislation. This study shows that risk assessment for a specific food safety hazard in a food cannot be undertaken individually, i.e., that different potential food safety hazards are linked and cannot be assessed in isolation. This is a weakness in current food safety risk assessment where individual hazards are risk rated separately. In this example, two food safety hazards, i.e., *Salmonella* contamination and ethylene oxide contamination, were associated with the same commodity, but that their presence/absence in a given food cannot be considered in isolation. As concerns rise over the potential for *Salmonella* contamination, individuals may decide to address that food safety hazard by utilizing ethylene oxide which, if it is then present above the MRL, becomes a potential food safety hazard in its own right. Guardianship in terms of risk analysis and risk management needs to consider separate food safety hazards both independently and in combination, as in driving the control and mitigation of one hazard in the supply chain, a second may then arise, a finding of importance in food safety governance.

Alongside the analysis of RASFF notifications, root cause analysis could benefit food regulators and industry by providing guidance on areas of focus for the prevention of incidents. Identifying the root causes of contamination or recall with the use of Ishikawa methodology (fishbone diagrams where causes are grouped into four categories of process, packaging, people, and place (environment)) could help to illustrate the background of an incident and develop risk mitigating strategies. Ishikawa analysis is particularly useful in a complex situation with multiple potential causes [88] and are likely to contribute to assessing whether *Salmonella* contamination of sesame seeds from India was interconnected with ethylene oxide incidents or not, and this creates room for further studies. Systematic analysis in assessing food safety incidents is increasing including the use of Functional Resonance Analysis Method (FRAM) see [89] and AcciMap frameworks [90].

## 5. Discussion

The Food Safety and Standards Authority of India (FSSAI) has quite a short history, being established in 2008 under the Food Safety and Standards Act of 2006 as a statutory body for laying down science-based standards for agri-food products. As the FSSAI embeds a food safety governance system, including the emergence of a science-based safety culture to ensure a risk-based approach to food safety and the protection of consumers, it will take time to build the capacity and infrastructure to reach a high level of food safety in practice [91]. This presents a vulnerability for any supply chain that includes raw materials from India, catalysing regulatory bodies and food businesses to consider their role and responsibilities within food governance and guardianship. There is potential for governance gaps to arise not only in ensuring product compliance, but also with developing information sharing infrastructure. When combined with the transition towards a risk-based regulatory approach in the EU (rather than a one size fits all approach), there is an onus on businesses to collaborate with regulators and share information to the benefit of public health. The rationale for developing collaboration between food business operators and regulators is primarily that industry has responsibility for the safety of the food they produce. In this particular example, the EU is a strategic trading partner for sesame seeds producers in India and has a role to play in ensuring the potential exporters and the Indian government are aware of the regulatory requirements that they must comply with.

Challenges can arise as multiple actors are involved in food safety governance and they can diverge in how they define problems, their individual and collective responsibilities in public health guardianship, and how they align their strategic intentions [92]. Promotion of good hygienic practice (GHP) and good manufacturing practice (GMP) within supply chains is important [93]. Another issue is to make efforts to build a strong food safety culture in order to benefit from knowledge, attitudes, values, motivation, and behaviour of food industry workers [94,95]. There is a crucial role, too, for “whistleblowers”, that is the employees themselves, to report any wrongdoing liable to lead to a serious risk to food safety. They can signal non-compliances or the need for testing provided there is appropriate regulatory protection of those who ‘blow the whistle’ and organizational culture across the supply chain facilitates employees disclosing their concerns [96,97].

Governments have the most control over the food supply chain at border crossings, whereas industry has the most control when receiving materials at premises [98]. The organization identifying a food safety problem can, in the case of positive release product checks, quarantine the product before it is placed on the market. Alternatively, if the organization operates a negative release protocol and after release to the market routine verification activities identify a non-conforming consignment, then the organization will have to implement a product withdrawal or full product recall [99]. This has been the case in this incident. Hence, the role of the company’s own checks is significant in strengthening food safety governance. Promoting collaboration among public and private stakeholders should improve the overall effectiveness and efficiency of the controls in place to assure public health. Although the European Commission strengthened border inspection point controls on sesame seeds from India due to the initial concern of the presence of ethylene oxide, this has not given rise to increased consignment failures at the EU borders. Instead, the surveillance undertaken by companies has driven the identification of non-compliant batches and the associated reactive activities in this public health incident. The following research question was considered in this study:

RQ1. What is the footprint of the ethylene oxide incident and what was the role of the ‘company’s own checks’ in the food safety governance processes in the 2020–2021 incident?

Whilst the ethylene oxide incident is still ongoing across a range of spices, herbs, and plant derived materials [66], the drivers of the 2020–2021 sesame seed incident are of interest. The publicly available sources of information show the extent of the outbreak and the interplay between the adoption of risk-based food safety regulation and associated hybrid models of food safety governance and guardianship between regulators and private organizations. The importance of company product testing rather than solely relying on testing as part of official regulatory controls is highlighted in this incident. Theory would suggest that public–private partnerships drive innovation in food governance structures [5], as well as greater agility and flexibility in governance activities when compared to purely regulatory governance approaches [6]. The key challenge is to integrate the efforts of public and private actors in the food supply chain in such a way as to achieve synergy and added value in terms of local and global food safety governance. One way to strengthen public–private food safety governance frameworks and to improve their effectiveness is to develop a joint root-cause analysis approach so that appropriate actions can be taken to prevent the recurrence of food incident. Indeed, the factors that have led to non-compliance are often related to working practices which result from management decisions [100]. Where guardianship fails in food supply chains, this is of particular concern. Another approach is to implement blockchain-based solutions in public–private models of food safety governance to provide transparency, visibility, and accountability in food supply chain [4]. Visibility offers what traceability cannot. Traceability is passive, following a product’s journey through the nodes of a supply chain; visibility enables food safety guardians to see what happens at each node, who is involved, and what the impacts are [101,102], increasingly in real-time. This makes it more difficult to embed supply chain practices that are prohibited by procurement standards and product specifications or indeed regulation. Thus, both traceability and visibility contribute to effective product recalls, but visibility additionally facilitates rapid identification of potential issues and effective crisis management.

The rationalities behind governments and food businesses activities are different (public interest vs. market gains), although, the final result should deliver the same outcome, i.e., ensuring food is consistently safe. The effective adoption of collaborative practices in food safety governance aligns the interests and expectations of all stakeholders involved [103]. The key to success is the collaboration of multiple players to create an inter-organizational guardianship network. Furthermore, various perspectives, values, and norms of actors involved offer a potential for the development of reflexive governance—a new mode of governance based on a collective learning process and opening paths towards new technologies and innovation [104]. Reflexive governance is “viewed as organizing a response to the risks by replacing traditional, hierarchical and deterministic governance approaches with more reflexive, flexible and interactive ones, which draw on diverse knowledge systems” [105] (p. 4). Reflexive governance has been developed to respond to complex risks that emerge from socio-ecological change—from nuclear disasters to climate change and food security [104]—thus it may be applied in the issues investigated in this study.

In the case of the ethylene oxide incident, the private actors developed a reactive form of sentinel system undertaking analysis once the concern was raised so they could identify clear signals of weak food safety governance. The recognition of these signals forms part of interconnected early warning systems that underpin the essential surveillance aspects of hybrid food safety governance approaches [106]. This has been considered previously, both in terms of food fraud activities that can cause harm such as illicit alcohol and methanol poisoning [107], and more generally with food safety [108]. Effective food safety guardianship requires the creation of a hierarchy of multiple signals that can collectively identify the existence and demonstrate the degree of a food safety concern [109]. Analysis of these signals to create early effective warning systems is coming ever closer as food supply chains digitize. Further, these guardianship systems need to be able to separate background noise in terms of surveillance data from the data or information signals that could show a guardianship or food safety governance failure [110]. This approach is worthy of further empirical research to study the role of private actors in food safety and food fraud signal detection, assessment, and risk communication within public–private food safety governance approaches. Dynamically growing numbers of food withdrawals and recalls associated with ethylene oxide and sesame seeds originating from India have caused loss of goodwill, brand value, a major food waste impact, and secession of supply, as well as a rise in raw material prices and finished products [111], highlighting the need for such governance approaches.

## 6. Conclusions

The consequences of the ethylene oxide incident are clear. One of the main contributions and novel findings of this article is the demonstration of the essential role of private actor self-surveillance within food safety governance systems, which will be a key aspect of risk-based regulation in the EU, and the adoption of new models of food supply system governance. It is worth noting that although the immediate problem with the presence of ethylene oxide in sesame seeds from India seems to be solved, the ethylene oxide recalls have spread to other foods and food ingredients. To verify supplier compliance with legal requirements, as part of their own due diligence obligations and increasingly as risk-based regulation is adopted across the EU, food business operators will need to adopt their own product assessment programs. These programs need to define the frequency of any product testing, and other associated verification methods, being determined by the organization’s risk characterization and risk assessment protocols as well as through their consideration of their development of their own, or collective signal detection, assessment, and risk communication activities. It is essential that food safety risk assessment is not seen as an assessment of individual hazards that could arise, but at a more systemic level, considering how contributing factors from a focus on one hazard can lead to another emergent risk occurring.

This study contributes to the literature on the adoption of risk-based food safety regulation and the associated value of developing collaborative hybrid models of food safety governance where both regulators and private organizations play a vital guardianship role in ensuring the health of the public. Whilst undertaking this study during the ethylene oxide incident might prove a limitation for in depth analysis, immediate learnings can be captured with regard to collaborative food safety governance. Collaboration in this instance is between EU regulators and business and between EU regulators and Indian food regulators, and Indian food regulators and Indian food businesses. The focus here on products of non-animal origin will inform private and public surveillance activities and how data are used from company testing and official regulatory controls to define the depth and breadth of a given public health issue at local levels, nationally and across Europe. This will help to ensure the food safety governance systems associated with plant-based foods minimize any risk to public health. As communities transition to a higher proportion of plant-based foods in their diet, the governance of products not of animal origin becomes of far more importance especially for cumulative carcinogens such as aflatoxins, and in this example, ethylene oxide.

## Figures and Tables

**Table 1:** Food withdrawals, recalls, and foodborne illness incidents associated with *Salmonella* and sesame seeds (2001–2021).

Year	Product	Pathogen	Country	Details	Sources
2001	Tahini used in halva imported from Turkey	*S. Typhimurium DT104*	Sweden Australia Norway	27 cases in Sweden; 18 cases in Norway; 14 cases in Australia	[31,33,37,38,45]
2002/2003	Tahini-halva from Egypt, Lebanon	*S. Montevideo* *S. Tennessee* *S. Orion*	Australia/New Zealand	Three outbreaks 68 cases	[31,36,40,41]
2004	Sesame seeds	*S. Mbandaka* *S. Virchow*	Serbia		[43]
2004	Sesame seeds	*Salmonella*	Australia	-	[42]
2010	Tahini	*Salmonella*	Australia	-	[46]
2012	Tahini used in hummus	*S. Bovismorbificans*	USA	Multistate outbreak 23 people in 7 states	[31,35,36]
2012	Tahini imported from Turkey	*S. Montevideo* *S. Maastrict* *S. Mbandaka*	New Zealand	One outbreak 27 people	[39]
2013	Hummus Tahini	*S. Montevideo* *S. Mbandaka*		16 cases; one hospitalization; one death	[34,36]
2013	Tahini	*Salmonella*	Canada	-	[47]
2018	Tahini	*Salmonella*	USA	-	[48,49]
2018	Tahini	*Salmonella*	Canada	-	[50]
2018	Tahini	*Salmonella*	USA	8 cases	[51]
2019	Tahini imported from Israel	*Salmonella*	USA	6 cases; 1 hospitalization	[52,53,54]
2019	Hummus	*Salmonella*	Ireland	-	[55]
2019	Tahini	*Salmonella*	UK	-	[56]
2019	Hummus	*Salmonella*	UK	-	[57]
2020	Sesame seeds from India	*Salmonella*	Ireland	-	[58]
2020	Tahini	*Salmonella*	Canada	-	[59]
2021	Tahini	*Salmonella*	USA	-	[60]
2021	Sesame oil	*Salmonella*	USA	-	[61]
2021	Halva	*Salmonella*	USA	-	[62]
2021	Hummus	*Salmonella*	USA	-	[63]
2021	Tahini	*Salmonella*	Canada	-	[64]
2021	Tahini	*Salmonella*	Canada	-	[65]

**Table 2:** Official controls sampling frequency for sesame seeds (2017–2020) (Source: Adapted from [82]).

Legislation	Country of Origin	Hazard	Frequency of Identity and Physical Checks (%)
EU 2017/186	India	*Salmonella*	20
EU 2019/1793	Ethiopia	*Salmonella*	50
EU 2019/1793	Nigeria	*Salmonella*	50
EU 2019/1793	India	*Salmonella*	20
EU 2019/1793	Sudan	*Salmonella*	20
EU 2020/1540	India	*Salmonella*	20
EU 2020/1540	India	Pesticide Residues	50

**Table 3:** Key search terms in the study.

Primary Term	Secondary Term
*Salmonella* AND	Sesame
	Seeds
	Tahini
	Helva
	Halva
	Hummus
	Hummus
	Hummus
Ethylene oxide AND	Risk
	Safety
	Maximum residual level or MRL
	*Salmonella*
	Sesame seeds

**Table 4:** Actions identified in the RASFF database following notifications linked to ethylene oxide contamination in sesame food products from India being issued over the period 1 September 2020–31 August 2021 (Source: Own elaboration based on [66]).

Action Identified in the Database	Number of Notifications
Informing the consignor	161
Informing the recipient(s)	75
Withdrawal from the market	60
Withdrawal from the recipient(s)	34
Recall from consumers	30
Monitoring of the recall/withdrawal	18
Public warning–press release	14
Re-dispatch	11
Official detention	11
Detained by the operator	9
Informing the authorities	7
Destruction	7
No stock left	5
Return to consignor	4
No action taken (no distribution from notifying country)	2
Placed under customs seals	1
Seizure	1
